# The association between headache and low back pain: a systematic review

**DOI:** 10.1186/s10194-019-1031-y

**Published:** 2019-07-15

**Authors:** Arani Vivekanantham, Claire Edwin, Tamar Pincus, Manjit Matharu, Helen Parsons, Martin Underwood

**Affiliations:** 1 University of Warwick, Coventry, UK; 2 University Hospitals Coventry and Warwickshire NHS Trust, Coventry, UK; 3 Centre for Epidemiology Versus Arthritis, University of Manchester, Manchester, UK; 4 Royal Holloway University of London, London, UK; 5 University College London (UCL) Queen Square Institute of Neurology, London, UK; 6 Warwick Clinical Trials Unit, Warwick Medical School, University of Warwick, Coventry, UK

**Keywords:** Primary chronic headaches, Persistent low back pain, Epidemiology, Chronic pain syndromes

## Abstract

**Background:**

To systematically review studies quantifying the association between primary chronic headaches and persistent low back pain (LBP).

**Main text:**

We searched five electronic databases. We included case-control, cross-sectional and cohort studies that included a headache and back pain free group, reporting on any association between persistent LBP and primary headache disorders. Methodological quality was assessed using Newcastle-Ottawa Scale. Our primary outcome was the association between primary headache disorders and persistent LBP. Our secondary outcomes were any associations between severity of LBP and severity of headache, and the relationship between specific headache sub-types classified as per International Classification of Headache Disorders (ICHD) criteria and persistent LBP.

We included 14 studies. The sizes of the studies ranged from 88 participants to a large international study with 404, 206 participants. Odds ratios for the association were between 1.55 (95% confidence interval (CI) 1.13–2.11) and 8.00 (95% CI 5.3–12.1). Study heterogeneity meant statistical pooling was not possible. Only two studies presented data investigating persistent LBP and chronic headache disorders in accordance with ICDH criteria.

**Conclusions:**

We identified a positive association between persistent LBP and primary headache disorders. The quality of the review findings is limited by diversity of populations, study designs and uncertainly about headache and LBP definitions.

**Trial registration:**

PROSPERO 2018 CRD42018086557.

**Electronic supplementary material:**

The online version of this article (10.1186/s10194-019-1031-y) contains supplementary material, which is available to authorized users.

## Introduction

Low back pain and headache are leading causes of disability worldwide [1, 2]. Each headache disorder has specific diagnostic criteria [3]. The commonest types of headache are migraine, tension-type (both primary headaches) and medication overuse headache (a secondary headache) [4]. Migraine and tension-type headache featured in two of the eight causes of chronic disease and injury, each affecting more than 10% of the world’s population [1]. Chronic headache is a severely disabling condition affecting around 3–4% of adults worldwide [5]. It is defined as a headache occurring on ≥15 days per month for more than 3 months [3].

Low back pain has a high healthcare burden, and in the most recent global burden of disease study, both low back pain and migraine were featured in the five leading causes of years lived with disability [1]. Around 4% of the UK population take time off work because of low back pain, resulting in around 90 million working days lost and between 8 and 12 million General Practitioner (GP) consultations per year [6]. Chronic low back pain is defined as pain felt in the area between the bottom of the rib cage and the buttock creases for more than 3 months [7].

There is a considerable focus in headache management in achieving a precise diagnosis in line with the International Classification of Headache Disorders [3]. Once a diagnosis is identified, management is focused accordingly [3, 8]. In contrast once serious causes of low back pain are excluded (malignancy, vertebral fractures, inflammatory disorders or infection) non-specific low back pain is diagnosed.^2, 3,^

People with persistent low back pain and people with chronic headache disorders are typically managed by clinicians from specific clinical specialities rather than experts in the management of chronic pain syndromes [9]. Whilst this approach may be appropriate for those living with one of these chronic conditions, it may be different if people have both. A previous systematic review of twin studies has identified a possible independent association between headache and low back pain [10]. People with both headache disorders and low back pain might constitute a neglected group that could have both conditions managed in combination rather than as separate entities. Here, we describe a systematic review of observational studies reporting the association between headaches (primary headaches, and chronic headaches) and persistent low back pain.

## Methods

We sought to identify all case control, cohort and cross-sectional studies reporting the relationship between primary headache disorders and persistent low back pain. We used a wide definition of headache disorders to reflect that exact headache type is often poorly defined and setting tight diagnostic criteria for study inclusion would exclude much of the available literature. Here, we use the term persistent to define low back pain duration rather than the term chronic [11]. This reflects that duration of low back pain is often poorly defined and using a strict definition for chronicity of back pain would exclude much of the available literature. For this review, we have reported the definitions of headache and low back pain used by the original authors of the included studies. This will allow the reader to interpret the findings cognisant of the definitions used.

The protocol for the review is registered on PROSPERO (PROSPERO 2018 CRD42018086557). This can be accessed via this link: http://www.crd.york.ac.uk/PROSPERO/display_record.php?ID=CRD42018086557.

### Searches

One reviewer (AV), with the assistance of an academic librarian, searched five electronic databases: Medline, Embase, Applied Social Sciences Index and Abstracts (ASSIA), PsychINFO and Web of Knowledge. These searches were supplemented by forward and backward citation tracking from included studies and relevant review papers. We excluded dissertations and conference proceedings. The electronic search terms included Medical Subject Headings (MeSH) headings, text words and truncation. Full details of the Medline search (as an exemplar) are available in online Appendix. Searches were run on 05/01/2018. A second search was run on 06/07/2018 to ensure recently published articles were included. The PRISMA 2009 checklist was used to ensure methodological quality [12]. The protocol for this systematic review was published on PROSPERO (registration number CRD42018086557).

### Primary outcome

The association between chronic headache disorders and persistent low back pain.

### Secondary outcomes

The relationship between severity of headache and low back pain.

The relationship between specific headache sub-types classified as per ICHD criteria and persistent low back pain [3].

We included any cross-sectional, cohort or case-control study, published in English. We sought to include studies reporting on the presence of headache, primary headache disorder, or medication overuse headache, with persistent low back pain with or without radicular pain. We had no restriction on age of participants. Studies were excluded if they did not compare participants to a control group ‘no headache or no back pain groups’.

### Screening

After removal of duplicates, two reviewers (AV and CE) screened all abstracts and titles against the eligibility criteria using Covidence systematic review software (Veritas Health Innovation, Melbourne, Australia). Full texts of potential studies were then screened by the same reviewers. A further reviewer (MU) arbitrate any disagreement and agreed the final eligible studies.

Two reviewers (AV and CE) independently assessed the methodological quality of each study using the Newcastle-Ottawa Quality Assessment Scale (for cohort and adapted for cross-sectional studies) [13], MU adjudicated disagreements. Studies were not excluded on the basis of quality. The Newcastle-Ottawa Assessment of bias measures quality according to a star-based system; there is separate scoring system for cohort and cross-sectional studies. Each study is judged on three categories, selection of study groups, comparability of groups, and the ascertainment of either the exposure of interest in case-control studies or outcome of interest in cohort studies. Good quality studies receive a minimum of six stars [14]. Authors’ conflicts of interests were noted.

### Analysis

The data on the association between headache and persistent low back pain were extracted independently by two reviewers (AV and CE) and then compared for the final data extraction table which was checked by a statistical reviewer HP. We extracted the descriptions of headache and back pain phenotypes as reported by the original authors. Where appropriate original authors were contacted for clarification. Due to study heterogeneity, statistical pooling was not appropriate. We therefore used a narrative synthesis approach. We extracted odds ratios from papers that reported the presence or absence of headache with the presence or absence of persistent low back pain. Some papers presented odds ratios from a variety of different statistical models; in these cases, we chose the odds ratio from the multivariate adjusted model. In other cases, we calculated or corrected odds ratios from the data presented within the paper. Results presented are from published data only.

## Results

### Searches

**Fig. 1:**
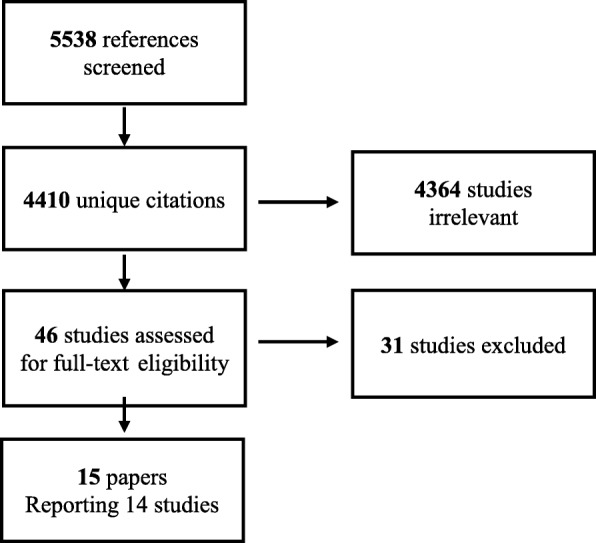
PRISMA Flowchart

We identified 5538 potentially relevant citations, which included 4410 unique citations, 4364 of which did not meet inclusion criteria. Overall, we identified 46 studies that warranted scrutiny of the full text, 15 of these were included in the review. Thirty-one studies were excluded because they did not include a headache and back pain free group, or did not report on any association between low back pain and headaches. We included 15 papers reporting on 14 studies (Fig. 1). Citation tracking did not yield any additional result.

## Study characteristics

Studies were heterogeneous, but a consistent positive association between headache and low back pain was found. This is consistent across countries, populations and study design but variable in magnitude. The odds ratios range from OR 1.72 (95% CI 1.38–2.15) [15, 16] to 8 (95% CI 5.3–12.1) [10].

Four studies were of children/adolescents (one cohort [17], three cross-sectional [18–20],) ten were adult studies (one cohort [21], four cross-sectional [10, 22–24], one cross-sectional older adult study [25], one cohort twin study [26], three cross-sectional twin studies) [15, 16, 27, 28] (Table 1).

**Table 1:** Study Characteristics

Study ID	Country	Inclusion/Exclusion Criteria	Definitions		Size of population (n)	Age	Male (%)
			Headache	Back pain			
Cross-sectional Studies							
Child and Adolescent Studies							
Ghandour et al., 2004 [15]	USA	Female adolescents (grades 6–10).	Self-reported headache.	Self-reported LBP	8370	12–16 years (range).	0 (0)
Sjolie et al., 2002 [16]	Norway	Pupils aged 14–16 years.	Self-reported weekly headache.	LBP, measured by a Nordic questionnaire.	88	14.1–16.1 years (range).	50 (57)
Swain et al., 2014 [17]	Europe, America, Israel)	Ages 11, 13, 15 years.	Self-reported headache.	Self-reported LBP.	404 206	9.8–17.3 years (range).	197 094 (49)
Adult Studies							
Ashina et al. 2018 [18]	Denmark	Ages 25–65 years from Danish Civil Registration System	Self-reported headache.	Self-reported LBP.	1300	49.1, 13.9 (mean, SD)	Not reported
Bejia et al. 2005 [19]	Tunisia	Staff at Fattouma Bourguiba Teaching Hospital.	Self-reported migraine.	Self-reported LBP.	350	37, 7.8 (mean, SD 7.88) 18–60 (range).	178 (51)
Bener et al., 2015 [20]	Qatar	Ages 15–65 years from primary care.	None given.	Roland-Morris Disability Questionnaire.	1829	15–65 years (range).	934 (51)
Yoon et al., 2013 [10]	Germany	Ages 18–65 years.	Self-reported headache.	Self-reported LBP.	9944	43, 13.1 (mean, SD).	4703 (47)
Older Adult Studies							
Ahangar et al. 2016 [21]	Iran	Ages > 60 years from Amirkola town.	Self-reported headache.	Self-reported LBP.	1499	Frequency of age ranges: 60–64 (36.9%) 65–69 (30%) 70–74 (17.6%) 75–79 (15%) 80–84 (6%) 85–99 (3%)	832 (55)
Twin Studies							
Hartvigsen et al., 2004 [22] (Hartvigsen et al., 2003 [23])	Denmark	Danish twin study.Twins aged > 75 years.	Self-reported migraine.	Self-reported LBP.	4484	70–102 years (range).	N/A
Hestbaek et al., 2004 [24]	Denmark	Danish twin study. Twin aged 12–22 years	Self-reported headache.	Self-reported LBP.Self-	9567	Frequency of age ranges: 12–13 (*n* = 1492). 14–16 (*n* = 2533). 17–19 (*n* = 2668). 20–22 (*n* = 2874).	4580 (49)
Schur et al., 2007 [25]	USA	Twins from the University of Washington Twin Registry (UWTR).	Self-reported a doctor’s diagnosis headache.	Self-reported a doctor’s diagnosis LBP.	3982	32.4, 14.7 (mean, SD).	1536 (39)
Cohort Studies							
Child and Adolescent Studies							
Jones et al., 2003 [26]	UK	Ages 11 to 14 years, from 39 secondary schools.	Self-reported headache.	Self-reported LBP.	933	11–14 years (range).	Not reported.
Adult Studies							
Angst et al., 2017 [27]	Switzerland	A representative age cohort of the general population of the canton of Zurich.	Self-reported headache.	Self-reported LBP.	4547	27/28 in 1986, then 49/50 in 2008.	1071
Twin Studies							
Hestbaek et al., 2006 [28]	Denmark	Danish twins born between: 1972–1982. Aged 12–22 years	Self-reported headache.	Nordic Back pain questionnaire.	Baseline: 9,600 (84%). Follow up: 6554.	17.27 (11–22) (mean, baseline). 17.38 (11–22) (mean, follow-up).	Baseline: 4654 (48%). Follow up: 2868 (44%).

1 LBP, Low back pain

Studies came from; Denmark (four studies) [15, 16, 22, 26, 27], USA (2 studies) [18, 28], and one each from Germany [10], Iran [25], Norway [19], Switzerland [21], Tunisia [23], UK [17], and Qatar [24]. One large multinational study included participants from 29 countries from Europe, North America and Israel [20, 28].

Study size ranged from 88 participants [19] to a large international study with 404,206 participants [20]. Three studies had 8,000–10,000 participants. Overall, we include data on studies with 460,195 participants. Ages ranged from 9.8 years [20] to 102 years [15, 16] (Table 1).

### Methodological quality assessment

All three cohort studies received seven stars or above [17, 21, 26]. Six cross-sectional studies were of good quality receiving seven stars or above [10, 15, 16, 18–20, 22, 24] (Table 2).

For each included study, details of study funding source and study authors’ conflicts of interests can be found in Additional file 1. Furthermore, the characteristics of excluded studies (for including the wrong outcome) can be found in Additional file 2.

**Table 2:** NOS for Cross-sectional and Cohort studies Quality Assessment using the Newcastle-Ottawa Scale

CROSS-SECTIONAL STUDIES	Selection				Comparability	Outcomes			TOTAL (maximum 10✯)	Overall Quality Assessment (AHRQ standards)
	Representiveness of exposed cohort	Sample Size	Non-respondents	Ascertainment of the exposure (risk factor)		Assessment of outcomes	Statistical test			
Child and Adolescent Studies										
Ghandour et al., 2004 [15]	✯	–	✯	✯	✯✯	✯✯	✯		8✯	Good
Sjolie et al., 2002 [16]	✯	–	✯	✯	✯✯	✯	✯		7✯	Good
Swain 2014 [17]	✯	✯	–	✯	✯✯	✯	✯		7✯	Good
Adult Studies										
Ashina et al. 2018 [18]	✯	–	✯	✯✯	✯✯	✯	✯		8✯	Good
Bejia et al. 2005 [19]	✯	–	–	✯	✯	✯	✯		5✯	Poor
Bener 2015 [20]	✯	✯	✯	✯	✯	✯	✯		7✯	Good
Yoon 2013 [10]	✯	–	–	✯✯	✯✯	✯	✯		7✯	Good
Older Adult Studies										
Ahangar 2016 [21]	–	✯	–	–	✯✯	✯	✯		5✯	Poor
Twin Studies										
Hartvigsen et al., 2004 (Hartvigsen et al., 2003) [22, 23]	✯	✯	✯	✯	✯✯	✯	✯		8✯	Good
Hestbaek et al., 2004 [24]	✯	–	–	✯	✯	✯	✯		5✯	Fair
Schur 2007 [25]	✯	–	–	✯	✯	✯	✯		5✯	Fair
COHORT STUDIES	Selection				Comparability	Outcomes			TOTAL (maximum 9✯)	Overall Quality Assessment (AHRQ standards)
	Representiveness of exposed cohort	Selection of non-exposed cohort	Ascertainment of exposure	Outcome not present at the start of the study		Assessment of outcomes	Length of follow-up	Adequacy of follow-up		
Child and Adolescent Studies										
Jones et al., 2003 [26]	✯	✯	–	✯	✯	✯	✯	✯	7✯	Good
Adult Studies										
Angst et al. 2017 [27]	✯	✯	✯	✯	✯✯	✯	✯	✯	9✯	Good
Hestbaek et al., 2006 [28]	✯	✯	–	✯	✯✯	✯	✯	✯	8✯	Good

**Table 3:** Summary of results of included studies

Study ID	Results	ORs (95% CI) for association between LBP and headache	n included in analysis
Cross-sectional Studies			
Child and Adolescent Studies			
Ghandour et al., 2004 [15]	In those with LBP more than once a week, 45% (CI 43.8–46.1) experienced headache more than once a week. Those with headaches more than once a week, 34% (CI 32.6–34.4) experienced LBP more than once a week.	2.1 (2.09–2.1) ^a^	n 8250
Sjolie et al., 2002 [16]	33 girls reporting LBP also had weekly headache. 22 boys reporting LBP experienced weekly headache.	2.5 (CI 0.8–8.0) LBP and weekly headache ^b^	n 82
Swain et al., 2014 [17]	12% of adolescents had LBP and headache. Univariate logistic modelling found adolescents with pain were at increased odds of experiencing co-existing pain.	2.9 (2.8–2.9)	n 404 206 n of headache and LBP 26 059
Adult Studies			
Ashina et al., 2018 [18]	Lifetime relative frequency of LBP was higher in individuals with any primary headache (migraine and/or tension type headache) than those with no primary headache 87% vs, 78%. There was a positive correlation with the number of days with tension type headache or migraine in past year and number of days with LBP in last year correlation *r 0.25, p < 0.001*. Logistic regression analysis of those with chronic headache with LBP, 33 (83%), those without LBP 7 (18%).	OR Primary headache including (migraine and tension type headache, with LBP) 1.7 (1.2–2.5). Episodic headache and LBP 1.7 (1.2–2.5). Pure migraine and LBP1.2 (0.7–2.1). Migraine and tension type headache 2.4 (1.3–4.4). OR Chronic headache and LBP 1.9 (0.8–4.5).	n 796
Bejia et al. 2005 [19]	In patients with LBP (*n* = 176), 29% had migraine. In patients with no LBP (*n* = 174), 14% of these had migraine.	2.55 (1.49–4.38) ^c^	n 350
Bener et al., 2015 [20]	LBP (*n* = 1034), 411 (40%) had headache, without LBP (*n* = 795), 198 (25%) had headache *p* < 0.001. Of those with LBP, 422 had it for < 6 weeks and 612 had it for >/= 6 weeks.	1.99 (1.62–2.44) ^d^	n 1034 LBP
Yoon et al., 2013 [10]	In 5605 who reported headaches in previous year, 255 had chronic headache and 5350 had episode headache. Migraine or those with migraine and including co-existing TTH diagnosed in 2933 respondents of whom 182 had chronic migraine and 2751 had episodic migraine. 76% of people with episodic headache had LBP, and 88% of those with chronic headache reported LBP. Chronic LBP occurred at higher rate in chronic versus episodic headache subtypes and at higher rate in migraine versus tension type headache subtypes. Frequent LBP among headache subtypes 8% of those with no headache, 15% of those episodic headaches, 57% of those with chronic headache reported frequent LBP.	OR LBP and episodic headache 3.8 (9 3.4–4.2) (n 8611) OR LBP and chronic headache 8 (5.3–12.1) eLBP and episodic migraine 4.8 (4.2–5.5) (n 6099) OR LBP and chronic migraine 9.3 (5.6–15.5) OR LBP and episodic migraine with no coexisting tension type headache 4.6 (4–5.4) (n 4940) OR LBP and chronic migraine with no coexisting tension type headache 9.5 (4.9–18.4) OR LBP episodic tension type headache 3.5 (3–4.1) (n 4524) OR LBP and chronic tension type headache 4.4 (2.1–9.2).	n 8611
Older Adult studies			
Ahangar et al. 2016 [21]	Headache independently associated with LBP. 51% with LBP had concomitant headache.	2.78 (2.22–3.49) ^f^	n with LBP 942
Twin Studies			
Hartvigsen et al., 2004 (Hartvigsen et al., 2003) [22, 23]	25% of participants (CI 95% 24–72%) experienced LBP in the last month. The prevalence was significantly different for men and women 20% (CI 18–22%) vs. 29% (CI 28–31%). 15% of those with LBP experienced migraine (females 15%, males 5%). OR of having LBP with migraine 1.72 (CI 1.38–2.15).	1.72 (1.38–2.15)	n 4484
Hestbaek et al., 2004 [24]	8210 of the cohort reported headache, 3704 reported LBP.	LBP > 30 days in past year (reference 0 days in past year) odds ratio 3.4 (2.32–4.98).	n in analysis not stated.
Schur et al., 2007 [25]	17% of participants reported high proportions of depression, LBP, and headache.	2.7 (CI 2.0–3.6)^g^	n 3937
Cohort Studies			
Child and Adolescent Studies			
Jones et al., 2003 [26]	A total of 8% of children (95% CI 5.8–9.2) reported headaches on 7 days during the month before the baseline survey. 933 children of 1046 who were LBP free at baseline participated at follow-up at median time 12.4 months (IQR 11.9–13.6), 168 reported LBP lasting > 1 day in the month before follow up survey.	1.82 (CI 1.04–3.18)^h^	n 933
Adult Studies			
Angst et al., 2017 [27]	344 subjects reported lumbar LBP, 101 lumbar LBP only, 303 cervical LBP, 243 reported both cervical and lumbar LBP. Lumbar LBP observed with headache (migraine/tension headache) OR 2.38 (CI 1.78–3.20)- moderate association. Cervical LBP also observed with headache (migraine/tension headache) odds ratio 2.62 (CI 1.94–3.53)	2.38 (CI 1.78–3.20)	n 499
Twin Studies			
Hestbaek et al., 2006 [28]	LBP reported in 3223 (34%). LBP- long 588 (6%)- 559 (95%) of which had headache, 134 (23%) of which had headache-long. Headache in 8266 (86%) of original sample (*n* = 9600)- 2974 (36%) had LBP, 559 (7%) LBP long, headache-long 591 (7%). Headache-long term- 591 (6%) of total sample of which 334 (57%) had LBP, 134 (23%) had LBP-long term. Multivariate logistic regression analysis was used to identify the association between health status at baseline and persistent LBP at follow-up. Results presented are the analysis of persistent LBP and persistent headache; model II included persistent LBP and persistent headache (LBP-long, headache- long). If persons in addition to persistent LBP in 1994 suffered from headache at al, the one-year prevalence of persistent LBP at follow-up was 27%, if headache was long lasting prevalence was 36%.	1.55 (1.13–2.11) (females) 2.4 (1.21–4.74) (males)	n 592

a OR in paper calculated by review statistician using weighted population estimates (raw data comorbidity not reported

b Review presents study model 3 results ordinal logistic regression analysis, model adjusting for frequency of physical activity, gender, each other, time spent on television/computer and BMI

c OR calculated by review statistician

d OR calculated by review statistician

e OR in paper corrected by review statistician

f Adjusted for age and sex, and adjusted odds ratio of chronic tension headache with concomitant LBP

g OR calculated by review statistician

h Model 3- adjusted for age, gender, smoking status, drinking status, education level, BMI

^1^ LBP, LBP, ^2^ OR, Odds ratios, ^3^ CI, Confidence intervals

### Definitions of headache and back pain

Definitions of low back pain varied. Due to the nature of observational studies low back pain was self-reported but the detail asked of participants, and described by studies was variable (Table 1). Swain et al. [20], used a five point scale to report low back pain frequently in the past 6 months. Hartvigsen et al. [15, 16], asked several specific questions regarding low back pain (‘have you during the past month suffered from back pain, acute low back pain or lumbago?’) Few studies measured self-reported back pain with established questionnaires. Hestbaek et al. 2006 [26], measured low back pain with a Nordic questionnaire as did Sjolie et al. [19],. Bener et al. [24], used a variety of outcome measures including the Roland-Morris Disability Questionnaire [29], a widely used health status measure for low back pain. Jones et al. [17], provided an illustration of a shaded area of the lower back participants where participants may have expected to experience low back pain in the past month. Angst et al. [21], distinguished between lumbar back pain versus cervical pain [21].

Persistency of low back pain was described infrequently. Chronic low back pain was defined by pain in the low part of the back and thigh pain radiating to above the knee lasting over 3 months by Bejia et al. [23] Hestbaek et al. 2006 [26], defined persistent low back pain being longer than 30 days in the past year, Yoon et al. asked for self-reported low back pain more than 15 days per month [10]. Ashina measured low back pain by self-reported frequency in the last year [22].

Like low back pain, there were varying definitions of headache, and chronic headache. Only the two studies by Ashina et al. [22], and Yoon et al. [10] used the ICHD (2nd edition) [30], now superseded by a third edition [3]. Yoon et al. also used a validated headache-screening questionnaire [10, 31]. Six studies specified that migraine or headache was self-reported [15, 16, 18–20, 23, 25]. Bener et al. [24], and Jones et al. [17], provided no definition, and Angst et al. [21] reported on participants reporting migraine or headache.

Hestbaek et al. [26], analysed participants according to any positive answers to headache regardless of aetiology (migraine, headache with nausea, headache with photophobia/phonophobia, severe ocular pain). Schur et al. [28] asked if participants had a doctor’s diagnosis of headache, the authors here also explained why they used a self-reported method rather than diagnostic criteria, as validated measures were too lengthy and diagnostic criteria were not agreed upon. Hartvigsen et al. [15, 16] asked participants in a survey whether a physician had ever told them they suffered from various diseases (outcome reported as migraine headache in results), answers were taken as valid if participants confirmed that a diagnosis was made by a physician. Therefore, whether participants are truly reporting migraine or another type of headache is unclear [15, 16].

Three studies involved face-to-face interviews within the sample. Ahangar et al. [25] used a mixture of questionnaires and in-person interviews to collect data from elderly people in the town of Amirkola, Iran. Bener et al. [24] used trained nurses to interview patients and complete questionnaires. Angst et al. [21] used a stratified subsamples of the original sample for face-to-face interviews, two-thirds of those participants scoring high risk for psychopathological syndromes according to the Symptom Checklist-90-R [32].

No studies reported on the severity of headache and severity of low back pain.

### Relationship between specific headache types and persistent low back pain

Table 3 summarises the results of the included studies. Ashina et al. [22] found 649/796 (81.5% of their participants had lifetime prevalence of low back pain); 321/796 (40.3%) of their participants had primary headache (migraine and/or frequent episodic tension type headache or chronic tension type headache). Of these 475/796 (59.7%) reported infrequent episodic tension type headache or no headache categorised as no headache in the past year; 281/796 (35.3%) had episodic headache, 80/796 (10.1%) had pure migraine, 138/796 (17.3% had pure tension type headache, and 103/796 (12.9%) had coexistent migraine and tension-type headache. No case of chronic migraine was identified in the study. Ashina et al. [22] noted a positive correlation between the number of days with tension type headache or migraine and number of days with low back pain in the past year (*r = 0.25, p < 0.001, r = 0.16, p < 0.001*, respectively). The lifetime relative frequency of low back pain was higher in those with primary headache migraine and or tension type headache than those without headache (87.2 versus 77.7%) (*p = 0.001*). The adjusted odds ratio for primary headache (migraine and or frequent tension type headache or chronic tension type headache) and low back pain was OR 1.7 (95% CI 1.2–2.5).

Yoon et al. [10], identified those affected by chronic low back pain and primary headache disorders. The outcome variable of low back pain was defined as presence of frequent low back pain (yes vs. no), defined as self-reported low back pain occurring on more than or equal to 15 days per month. We present their multivariate analysis results (adjusted for age, gender, smoking status, drinking status, education level, BMI).

The association between migraine and low back pain was OR 1.2 (95% CI 0.7–2.1) (Ashina et al.) [22], and frequent low back pain and episodic migraine (no coexisting tension-type headache) OR 4.6 (95% CI 4.0–5.4)(Yoon et al) [10]. The association between episodic headache and low back pain was OR 1.7 (95% CI 1.2–2.5) (Ashina et al.) [22] and frequent low back pain and episodic headache OR 3.8 (95% CI 3.4–4.2) (Yoon et al) [10]. Associations between tension-type headache and low back pain were as follows; pure tension-type headache and low back pain OR 1.9 (95% CI 1.2–3.1) (Ashina et al.) [22] and episodic tension-type headache OR 3.5 (95% CI 3.0–4.1) (Yoon et al) [10].

### Combining migraine and tension type headache disorders and experience of low back pain

Ashina et al. [22] found an OR 2.4 (95% CI 1.3–4.4), and Yoon et al. [10] found an OR 4.8 (95% 4.2–5.5) between frequent low back pain and episodic migraine with coexisting tension-type headache.

### Chronic headache and low back pain

Ashina et al. [22] found 40 individuals that reported chronic headache. In their analysis of chronic headache and low back pain the adjusted odds ratio was 1.9 (95% CI 0.8–4.5). Low back pain was used in analysis if it had been reported occurring at least 1 day in the past year.

Yoon et al. [10], found an odds ratio of chronic low back pain and chronic headache of 8 (95% CI 5.3–12.1); low back pain and chronic migraine with coexisting tension type headache OR 9.3 (95% CI 5.6–15.5); low back pain with chronic migraine (no coexisting tension type headache) OR 9.5 (95% CI 4.9–18.4); low back pain and chronic tension type headache OR 4.4 (95% CI 2.1–9.0).

## Discussion

In this review, we aimed to identify and describe an association between primary headache disorders and persistent low back pain. These are conditions that generate considerable morbidity globally,^1^ [33], but that have not been widely managed as co-morbidities. We have identified 14 studies reporting an association between primary headache disorders and persistent low back pain. Overall, there was a positive association in all studies, with odds ratios estimating the relationship between primary headache and low back pain ranged from 1.55 (95% CI 1.13–2.11) and 8.00 (95% CI 5.3–12.1). This appears to be a consistent finding irrespective of population studied or the study design. Although methodological quality varied, the larger epidemiological studies of Swain and Yoon are likely to illustrate a true population association due to the large population covered [10, 20]. Although, because of the extreme heterogeneity of study designs, definitions and populations studied, we cannot provide a pooled estimate the results indicate that a relationship exists between headache and low back pain. Thus, indicating reasonable grounds for further study.

We have followed the PRISMA guidance for systematic reviews during this review process [12]. The studies we identified had a large range of number of participants, a variety of methodologies, a large variety of countries, represented and participants of all ages. The total sample size represented in this review was 460 435. The review is limited by the varying definitions of chronic headache and low back pain, and in many cases, there was little explanation as to what was taken as a case definition of either headache (chronic or otherwise) or low back pain (Table 1). Some studies reported migraine specifically, but this was self-reported diagnosis, therefore may include non-migrainous headache subtypes [15, 16]. That no studies used current diagnostic or classification criteria of headache constrains the inferences that can be drawn from the existing literature. We looked for the association between chronic headache disorders and persistent low back pain but chronicity was not always determined in the published literature, and where chronicity was clarified the definitions used were variable.

Our review may be susceptible to publication bias. Attempts at eliminating bias in observational studies have been made, including the STROBE guidelines, which provides a checklist for authors of observational studies to use when disseminating data and result [34]. This includes advice on reporting all outcome events or measures, which should therefore include negative results. However, despite a well-designed and a comprehensive search strategy in a systematic review, positive outcomes are more likely to be published than negative outcomes [35]. Since our initial scoping review indicated that data would be sparse we have included all published studies and not set a size or quality criterion to define study inclusion. Data published in languages other than English may also have been missed, however, given the consistency of our findings it is unlikely that any such studies will materially affect our overall conclusions.

We are constrained by the variable approaches to define headache disorders by the original authors. Nevertheless, we identified an important association between chronic headache and persistent low back pain in the two studies that used ICHD diagnostic criteria [3, 30] to define chronic headache and formally differentiate between primary headache disorders. Yoon et al. used ICHD to diagnose the primary headache disorders and chronic headache disorders; they also used our preferred criteria of diagnosing low back pain [10]. Ashina et al. also used ICHD criteria for headache diagnosis.^10^ [22], A combined approach to treating both conditions could reduce pill-burden, medication overuse, and provide a holistic treatment approach for two chronic pain syndromes.

A large-scale European study found moderate to severe chronic pain reported in 19% of adults, notably half of these felt their pain was inadequately managed [33] A number of countries have recognised the therapeutic benefits of less location-specific pain relief in recent evidence-base back pain management guidelines (including cognitive behavioural therapies and mindfulness training) [9, 36, 37]. Treating chronic pain remains a challenge for clinicians and unfortunately unrealistic expectations of short-term pain control can lead to pharmacological over prescription (opioids in particular), and even excess interventions (including surgery or nerve blockade) [38]. Although 90% of patients who consult their general practitioner for low back pain in the UK stop consulting within 3 months, most are still experiencing low back pain and related disability 1 year after consultation [39]. This suggests that either those affected feel that there is limited help available, or that for another reason consultation is not worthwhile [40].

Understanding chronic pain is complex. There is some evidence suggesting that chronic pain conditions can be exacerbated by changes in psychological processing [41]. A review article surmised that chronic pain can lead to a vicious cycle of progressive changes in psychological well-being which are aggregated by reduced dopaminergic effects, pain sensitisation leading to worsening pain states through anti-reward and stress related neuroadapations [41]. Another low back pain study found that people exhibiting deficits in emotional awareness or difficulties in processing feelings had higher odds of experiencing low back pain [42]. There is also a genetic overlap between migraine and depressive illness [43].

Recognition of the impact that psychological processing can have on chronic pain has been recognised by the use of non-pharmacological treatments in chronic pain management. Examples include exercise therapy, and psychological therapies such as cognitive behavioural therapy [44]. Behavioural therapies have been endorsed by American Headache society, who describe chronic headache as a biopsychosocial disorder, involving not just physical disease but recognising the potential contribution of psychological processing and stressors to chronic headache symptomology [45].

In addition to considering psychosocial determinants of chronic pain processing in persistent low back pain and chronic headache, there are possible biological explanations. A previous systematic review on low back pain and other comorbidities has suggested that differences in gene expression could be implicated in the process of pain perception and signalling [46]. Similarly in migraine research, certain genetic mutations have been linked with differences in pain sensation [47]. For example, mutations in NGF (nerve growth factor) causing loss of pain perception, and TRPM8 (transient receptor potential cation channel subfamily M member 8) a receptor-activated non selective cation channel involved in sensing cold and possibly modulating pain sensation [47]. Monoclonal antibodies targeting calcitonin gene- related peptide (CGRP) have also been shown to have some effect in migraine prophylaxis [48] CGRP has been related to other pain mechanisms, including facet joint pain, which is a recognised cause and contributor to low back pain. A 2017 systematic review on CGRP reported that numerous studies have found an association between measured CGRP levels and pain, concluding that CGRP could be a neuromodulator in pain syndromes other than migraine [49]. Indeed, CGRP fibres have been found in degenerated human intervertebral discs sensory nerve fibres, further suggesting it may a role in nociceptive back-related pain [50]. Therefore, it is biologically plausible that the neuromodulator CGRP is involved in an association between low back pain and migraine. In this review one study in particular, Yoon et al., identified a particularly strong association between migraine and chronic low back pain [10]. This lends some support to the notion that there might be a particular association between migraine and persistent back pain mediated through a specific biological mechanism.

Headache disorders, including migraine, may be associated with chronic painful disorders other than back pain. For example, the prevalence of co-morbid migraine in people with fibromyalgia has ranged from 18% to 36% in different studies [51]. There is a plausible link for this association through the role of central sensitisation in both disorders [51]. We are not aware of any systematic reviews quantifying the strength of the association between fibromyalgia and headache disorders. Further epidemiological work is needed to more fully explore the interrelationship between headache disorders and other painful disorders.

## Conclusion

Further study should focus on the prospective and observational study of chronic headache patients and experience of persistent low back pain. In addition, study designs that examine apparent mechanisms that account for an association between persistent low back pain and primary headache disorders, in particular chronic headache would be beneficial. Such studies could lead us closer to finding concomitant management of both pain syndromes.

## Clinical implications

- Low back pain and headache are significant causes of disability worldwide.
- People with both low back pain and headache disorders may constitute a neglected patient group that could have both conditions managed in combination, rather than as separate entities.
- We identified a positive association between low back pain and headache disorders.
- A combined approach to treating both conditions could reduce pill-burden, medication overuse and provide a holistic treatment approach for the two chronic pain syndromes.

### Additional files

Additional file 1: Conflicts of interests and funding of included studies. (DOCX 15 kb)

Additional file 2: Characteristics of excluded studies for wrong outcome. (DOCX 18 kb)

## Data Availability

The data used and analysed during this review are available from the corresponding author on reasonable request.

## Appendix

### MEDLINE search strategy

The search strategy for MEDLINE is included below.

This was adapted for the other databases.

1. Headache/(28156)
2. exp. Migraine Disorders/(28066)
3. exp. headache disorders, primary/(32171)
4. exp. Headache Disorders/(35499)
5. migraine*.mp. (36142)
6. medication overuse.mp. or exp. Prescription Drug Overuse/(1150)
7. headache.mp. or exp. Headache/(74812)
8. 6 and 7 (942)
9. exp. Tension-Type Headache/(2081)
10. headache*.mp. (82863)
11. 1 or 2 or 3 or 4 or 5 or 8 or 9 or 10 (101688)
12. exp. Back Pain/ or exp. Low Back Pain/(37275)
13. (back pain or low back pain or backpain or low backpain or back-pain or low back-pain).mp. [mp=title, abstract, original title, name of substance word, subject heading word, keyword heading word, protocol supplementary concept word, rare disease supplementary concept word, unique identifier, synonyms] (50956)
14. 12 or 13 (51111)
15. exp. Cohort Studies/or cohort.mp. (2032295)
16. exp. Case-Control Studies/or case control*.mp. or case-control*.mp. [mp=title, abstract, original title, name of substance word, subject heading word, keyword heading word, protocol supplementary concept word, rare disease supplementary concept word, unique identifier, synonyms] (1024081)
17. exp. Epidemiologic Methods/or epidemiol*.mp. (6107794)
18. (cross section* or cross-section*).mp. [mp=title, abstract, original title, name of substance word, subject heading word, keyword heading word, protocol supplementary concept word, rare disease supplementary concept word, unique identifier, synonyms] (378101)
19. 15 or 16 or 17 or 18 (6209092)
20. 11 and 14 and 19 (1125)
21. limit 20 to english language (1015)

## Abbreviations

ASSIA: Applied Social Sciences Index and Abstracts
CGRP: Calcitonin Gene-Related Peptide
CI: Confidence Interval
GP: General Practitioner
ICHD: International Classification of Headache Disorders
LBP: Low back pain
MeSH: Medical Subject Headings
TRPM8: Transient Receptor Potential cation channel subfamily M member 8
