# Supplementary material for: The association between headache and low back pain: a systematic review
Source: J Headache Pain. 2019 Jul 15;20(1):82. doi: 10.1186/s10194-019-1031-y (PMC6734435; doi:10.1186/s10194-019-1031-y)
Supplement: Supplementary file 1 — Conflicts of interests and funding of included studies. (DOCX 15 kb) [file 10194_2019_1031_MOESM1_ESM.docx]

**APPENDIX B:** **Conflicts of interests and funding of included studies**

| **Conflicts of interests and funding of included studies** | | |
| --- | --- | --- |
| **Study ID** | **Conflicts of Interests** | **Funding** |
| Ahangar, 2016 | None declared. | Finance from faculty of medicine, Babol university of medical sciences. |
| Angst, 2017 | None declared. | Funding grand no 3200-050881.97/1 and 32/50881.97 of Swiss National Science Foundation. Supported by Zurzach Rehabilitation Foundation SPA, Bad Zurazach, Switzerland. |
| Ashina, 2018 | Ashina received honoraria for lecturing and serving as a consultant for Allergan, for serving as a consultant for Amgen and for lecturing for Avanir Pharmaceuticals. R. Lipton has received grant support from the National Institutes of Health, the National Headache Foundation and the Migraine Research Fund. He serves as consultant, serves as an advisory board member or has received honoraria from Alder, Allergan, American Headache Society, Autonomic Technologies, Boston Scientific, Bristol Myers Squibb, Cognimed, CoLucid, Eli Lilly, eNeura Therapeutics, Merck, Novartis, Pfizer and Teva, Inc. Lars Bendtsen serves on the scientific advisory board for Biogen. D. Buse has received grant support and honoraria from Allergan, Amgen, Avanir and Eli Lilly, and has received research support funded by Allergan, Alder, Avanir, CoLucid, Dr. Reddy's, and Labrys via grants to the National Headache Foundation and/or Montefiore Medical Center. R. Jensen has received honoraria for lectures and patient leaflets from MSD, Berlin‐Chemie Menarini, ATI and Pfizer and served on medical advisory boards for LindeGas, ATI and Electrocore. A.C. Lyngberg and N. Hajiyeva have no disclosures. | East Denmark Health Science Research Forum, the Danish Medical Association Research Fund, the Danish Health Insurance Foundation, the Danish Hospital Foundation for Medical Research, the Danish Headache Society, the Cool Sorption Foundation, GlaxoSmithKline A/S, Merck Sharp Dohme A/S, Pfizer A/S, Lundbeck Pharma A/S and H. Lundbeck A/S. The funding sources have not been involved in the conduct of the study. |
| Bejia, 2004 | None declared. | - |
| Bener, 2015 | None declared. | Qatar Foundation- funding. Hamad Medical Corporation for support and granting ethical approval. |
| Ghandour , 2004 | None declared. | - |
| Hestbaek, 2004 | No commercial conflicts. | L.H. funded by foundation for chiropractic research and postgraduate education, Denmark. |
| Hestbaek, 2006 | No competing interests. | L.H. funded by foundation for chiropractic research. |
| Hartvigsen, 2002/2003 | No benefits from commercial party. | US National Institute on Aging Research Grant NIA-POI-AG08761. Danish research councils. Danish National Research Foundation. Federal/foundation funds received. |
| Jones, 2003 | None declared. | Funding Colt Foundation. MRC PhD studentship. |
| Schur 2007 | None declared. | - |
| Sjolie, 2002 | None declared. | - |
| Swain 2014 | None declared. | Health Behaviour in School Aged Children collaboration with WHO/EURO. Funding data collection funded at a national level. National Health Service Scotland and Chief Medical Officer Directorate of Scottish Government funded the Health Behaviour in School Aged children International coordinating centre at child and adolescent health research unit, University of Edinburgh. |
| Yoon, 2013 | A.M. employee for and holds stocks in Allergan Inc. M.O. scientific support and/or honaria from Biogen Idec, Novartis, Sanofi-Aventis, Pfizer, Teva. Research grants, German ministry for education and research. H-C.D consultant for and member of speakers bureau Allergan Inc., Zaza Katsarava grants and research support form Allergan, Inc. | Allergan Inc. funded imprint publication science, New York. Funded by German Federal Ministry for Education and Research. |
