# Supplementary material for: The association between headache and low back pain: a systematic review
Source: J Headache Pain. 2019 Jul 15;20(1):82. doi: 10.1186/s10194-019-1031-y (PMC6734435; doi:10.1186/s10194-019-1031-y)
Supplement: Supplementary file 2 — Characteristics of excluded studies for wrong outcome. (DOCX 18 kb) [file 10194_2019_1031_MOESM2_ESM.docx]

## APPENDIX C: Characteristics of excluded studies for wrong outcomes

| **Characteristics of excluded studies for wrong outcomes** | | | |
| --- | --- | --- | --- |
| **Study ID** | **Sample size** | **Country** | **Explanation** |
| Adamson, G.; Murphy, S.; Shevlin, M.; Buckle, P.; Stubbs, D.  Profiling schoolchildren in pain and associated demographic and behavioural factors: a latent class approach  Pain 2007;129(3):295-303  2007 | 679 | United Kingdom | Did not specifically report on presence or absence of low back pain and presence or absence of headache. |
| Ajdacic-Gross, V.; Horvath, S.; Canjuga, M.; Gamma, A.; Angst, J.; Rossler, W.; Eich, D.  How ubiquitous are physical and psychological complaints in young and middle adulthood?: A longitudinal perspective  Social Psychiatry and Psychiatric Epidemiology 2006;41(11):881-888  2006 | 4547 | Switzerland | Did not specifically report on presence or absence of low back pain and presence or absence of headache. |
| Anttila, P.; Metsahonkala, L.; Mikkelsson, M.; Helenius, H.; Sillanpaa, M.  Comorbidity of other pains in schoolchildren with migraine or nonmigrainous headache  J Pediatr 2001;138(2):176-80  2001 | 622 | Finland | Compared migraine with non-migranous headache, but no non-headache group. |
| Armenian, H. K.; Halabi, S. S.; Khlat, M.  Epidemiology of primary health problems in Beirut  J Epidemiol Community Health 1989;43(4):315-8  1989 | 2752 households. | Lebanon | Did not specifically report on presence or absence of low back pain and presence or absence of headache. |
| Assadeck, H.; Toudou Daouda, M.; Hassane Djibo, F.; Douma Maiga, D.; Adehossi Omar, E.  Prevalence and characteristics of chronic pain: Experience of Niger  Scandinavian Journal of Pain 2017;17():252-255  2017 | 1927 | Niger | Did not specifically report on presence or absence of low back pain and presence or absence of headache. |
| Baek, C.; Park, J. B.; Lee, K.; Jung, J The association between Korean employed workers' on-call work and health problems, injuries  Annals of Occupational and Environmental Medicine 2018;30 (1) (no pagination)(19):  2018 | 290246 | Korea | Did not specifically report on presence or absence of low back pain and presence or absence of headache. |
| Barke, A.; Gassmann, J.; Kroner-Herwig, B.  Cognitive processing styles of children and adolescents with headache and back pain: A longitudinal epidemiological study  Journal of Pain Research 2014;7():405-414  2014 | 6400 | Germany | Did not specifically report on presence or absence of low back pain and presence or absence of headache. |
| Bener, A.; Verjee, M.; Dafeeah, E. E.; Falah, O.; Al-Juhaishi, T.; Schlogl, J.; Sedeeq, A.; Khan, S.  Psychological factors: Anxiety, depression, and somatization symptoms in low back pain patients  Journal of Pain Research 2013;6():95-101  2013 | 2742 | Qatar | Did not specifically report on presence or absence of low back pain and presence or absence of headache. |
| Bener, Abdulbari; Dafeeah, Elnour Elnaeim; Alnaqbi, Khalid  Prevalence and correlates of low back pain in primary care: what are the contributing factors in a rapidly developing country  Asian spine j 2014;8(3):227-36  2014 | 1829 | Qatar | Did not specifically report on presence or absence of low back pain and presence or absence of headache. |
| Bingefors, K.; Isacson, D.  Epidemiology, co-morbidity, and impact on health-related quality of life of self-reported headache and musculoskeletal pain--a gender perspective  Eur J Pain 2004;8(5):435-50  2004 | 8000 | Sweden | Did not specifically report on presence or absence of low back pain and presence or absence of headache. |
| Brattberg, G.  The incidence of back pain and headache among Swedish school children  Qual Life Res 1994;3 Suppl 1():S27-31  1994 | 1245 | Sweden | Did not specifically report on presence or absence of low back pain and presence or absence of headache. |
| Brattberg, G.  Do pain problems in young school children persist into early adulthood? A 13-year follow-up  Eur J Pain 2004;8(3):187-99  2004 | 335 | Sweden | Did not specifically report on presence or absence of low back pain and presence or absence of headache. |
| Brattberg, G.  Back pain and headache in Swedish schoolchildren: A longitudinal study  Pain Clinic 1993;6(3):157-162  1993 | 450 | Sweden | Did not specifically report on presence or absence of low back pain and presence or absence of headache. |
| Brattberg, G.; Wickman, V.  Prevalence of back pain and headache in Swedish school children: A questionnaire survey  Pain Clinic 1992;5(4):211-220  1992 | 1245 | Sweden | Did not specifically report on presence or absence of low back pain and presence or absence of headache. |
| Brochet, B.; Michel, P.; Barberger-Gateau, P.; Dartigues, J. F.; Henry, P.  Pain in the elderly: An epidemiological study in south-western France  Pain Clinic 1992;5(2):73-79  1992 | 2792 | France | Did not specifically report on presence or absence of low back pain and presence or absence of headache. |
| Christensen, J. O.; Johansen, S.; Knardahl, S.  Psychological predictors of change in the number of musculoskeletal pain sites among Norwegian employees: a prospective study  BMC Musculoskeletal Disorders 2017;18 (1) (no pagination)(140):  2017 | 2989 | Norway | Did not specifically report on presence or absence of low back pain and presence or absence of headache. |
| Csupak, B.; Sommer, J. L.; Jacobsohn, E.; El-Gabalawy, R.  A population-based examination of the co-occurrence and functional correlates of chronic pain and generalized anxiety disorder  Journal of Anxiety Disorders 2018;56():74-80  2018 | 25 113 | Canada | Did not specifically report on presence or absence of low back pain and presence or absence of headache. |
| Dartigues, J. F.; Michel, P.; Lindoulsi, A.; Dubroca, B.; Henry, P.  Comparative view of the socioeconomic impact of migraine versus low back pain  Cephalalgia 1998;18 Suppl 21():26-9  1998 | 20 625 | France | Did not specifically report on presence or absence of low back pain and presence or absence of headache. |
| Dorner, T. E.; Stein, K. V.; Hahne, J.; Wepner, F.; Friedrich, M.; Mittendorfer-Rutz, E.  How are socio-demographic and psycho-social factors associated with the prevalence and chronicity of severe pain in 14 different body sites? A cross-sectional population-based survey  Wiener Klinische Wochenschrift 2018;130(1-2):14-22  2018 | 15 474 | Austria | Did not specifically report on presence or absence of low back pain and presence or absence of headache. |
| Duckro, P. N.; Schultz, K. T.; Chibnall, J. T.  Migraine as a sequela to chronic low back pain  Headache 1994;34(5):279-81  1994 | 46 | United States | Did not specifically report on presence or absence of low back pain and presence or absence of headache. |
| El-Metwally, A.; Salminen, J. J.; Auvinen, A.; Macfarlane, G.; Mikkelsson, M.  Risk factors for development of non-specific musculoskeletal pain in preteens and early adolescents: a prospective 1-year follow-up study  BMC Musculoskelet Disord 2007;8():46  2007 | 1756 | Finland | Did not specifically report on presence or absence of low back pain and presence or absence of headache. |
| Fernandez-de-las-Penas, C.; Hernandez-Barrera, V.; Alonso-Blanco, C.; Palacios-Cena, D.; Carrasco-Garrido, P.; Jimenez-Sanchez, S.; Jimenez-Garcia, R.  Prevalence of neck and low back pain in community-dwelling adults in Spain: a population-based national study  Spine 2011;36(3):E213-9  2011 | 43 072 | Spain | Did not specifically report on presence or absence of low back pain and presence or absence of headache. |
| Fujii, T.; Oka, H.; Katsuhira, J.; Tonosu, J.; Kasahara, S.; Tanaka, S.; Matsudaira, K.  Association between somatic symptom burden and health-related quality of life in people with chronic low back pain  PLoS ONE 2018;13 (2) (no pagination)(e0193208):  2018 | 3100 | Japan | Did not specifically report on presence or absence of low back pain and presence or absence of headache. |
| Gaul, C.; Schmidt, T.; Czaja, E.; Eismann, R.; Zierz, S.  Attitudes towards complementary and alternative medicine in chronic pain syndromes: a questionnaire-based comparison between primary headache and low back pain  BMC Altern Med 2011;11():89  2011 | 432 headache, 194 low back pain | Germany | Did not specifically report on presence or absence of low back pain and presence or absence of headache. |
| Hartvigsen J.; Christensen K.; Frederiksen H.; Petersen HC.; Pedersen HC.  Genetic and environmental contributions to back pain in old age: a study of 2,108 danish twins aged 70 and older.  Spine Apr 2004;29(8):897-901; discussion 902  2004 Apr | 2108 | Denmark | Did not specifically report on presence or absence of low back pain and presence or absence of headache. |
| Kroner-Herwig, B.; Gorbunova, A.; Maas, J.  Predicting the occurrence of headache and back pain in young adults by biopsychological characteristics assessed at childhood or adolescence  Adolescent Health, Medicine and Therapeutics 2017;8():31-39  2017 | 1522 | Germany | Did not specifically report on presence or absence of low back pain and presence or absence of headache. |
| Page, M. G.; Fortier, M.; Ware, M. A.; Choiniere, M.  As if one pain problem was not enough: Prevalence and patterns of coexisting chronic pain conditions and their impact on treatment outcomes  Journal of Pain Research 2018;11():237-254  2018 | 3966 | Quebec | Did not specifically report on presence or absence of low back pain and presence or absence of headache. |
